# Plaies trachéales post intubations dans la chirurgie cervicale: à propos de 3 cas

**DOI:** 10.11604/pamj.2017.28.168.12792

**Published:** 2017-10-20

**Authors:** Ndeye Fatou Thiam, Evelyne Siga Diom, Cire Ndiaye, Abdou Sy

**Affiliations:** 1 Service d’ORL, CCF, Hôpital Militaire de Ouakam, Dakar, Sénégal; 2 Service d’ORL-CCF, Hôpital Fann, Dakar, Sénégal

**Keywords:** Intubation, thyroïdectomie, chirurgie cervicale, Intubation, thyroidectomy, cervical spine surgery

## Abstract

Nous rapportons 3 cas rares de plaies trachéales post-intubations découvertes au cours de chirurgie cervicale. Il s’agissait de 3 patientes âgées de 27, 30 et 42 ans admises au bloc opératoire pour une thyroïdectomie totale sous anesthésie générale indiquée devant un goitre hétéro-multi-nodulaire. L’intubation était orotrachéale à l’aide d’un guide rigide avec une sonde N°7,5 munie d’un ballonnet. Nous avons découvert en per opératoire une protrusion du ballonnet dans le champ opératoire à travers une effraction trachéale postéro-latérale. Le traitement avait consisté en une fermeture de la brèche trachéale dans 2 cas et une abstention chirurgicale dans le troisième cas suivi d’un drainage de la loge thyroïdienne. Les suites opératoires étaient simples. Les plaies trachéales post-intubations sont rares. Leur découverte per opératoire au cours d’une chirurgie cervicale est exceptionnelle. Les causes sont multiples. Il s’agit des difficultés d’intubation, d’une déchirure de la membraneuse par le bec de la sonde d’intubation, par un guide rigide, un effort de toux avec ballonnet gonflé au réveil, un sur-gonflage du ballonnet, de la modification structurale et anatomique de la trachée dans les goitres anciens. Il n’y a pas de consensus dans le traitement.

## Abstract (en)

We report 3 rare cases of post-intubation tracheal lacerations detected during cervical spine surgery. Our study involved 3 patients aged 27, 30 and 42 years admitted to the operating room for total thyroidectomy for heterogeneous multinodular goitre under general anesthesia. Orotracheal intubation using 7.5 rigid guide probe and balloon cuff was performed. During surgery we discovered a protrusion of the balloon cuff at the surgical site through posterolateral tracheal rupture. The treatment was based on closure of the tracheal rupture in 2 cases and on surgical abstention in the third case, followed by a drainage of the thyroid lodge. The postoperative course was uneventful. Post-intubation tracheal lacerations are rare. Intraoperative detection during cervical spine surgery is exceptional. They have multiple causes: intubation difficulties, membranous rupture caused by probe beak, by a rigid guide, coughing effort with inflated balloon at the awakening, balloon hyperinflation, structural and anatomical changes of the trachea in patients with chronic goiter. There is no consensus about treatment.

## Introduction

Les plaies trachéales post-intubations sont rares et souvent méconnues. En moyenne, 1 intubation sur 40 000 en chirurgie courante se complique d’une plaie trachéale [1]. Il s’agit d’un accident grave pouvant engager le pronostic vital du fait surtout des troubles respiratoires et d’autres conséquences qu’elles peuvent induire. Aussi, il n’y a pas de consensus dans la prise en charge de ces plaies. Peu de cas ont été retrouvés dans la littérature. Nous rapportons 3 cas de plaie trachéale post-intubation découverts au cours de chirurgie thyroïdienne, avec apparition spectaculaire du ballonnet dans le champ opératoire.

## Patient et observation

**Figure 1:**
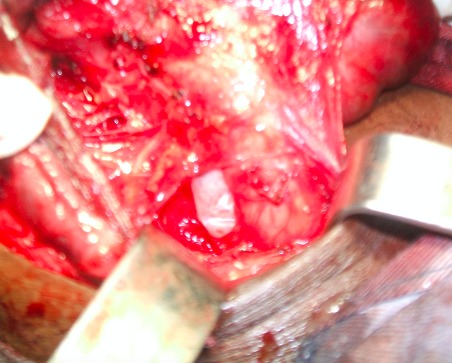
Brèche trachéale d’environ 2,5cm

**Figure 2:**
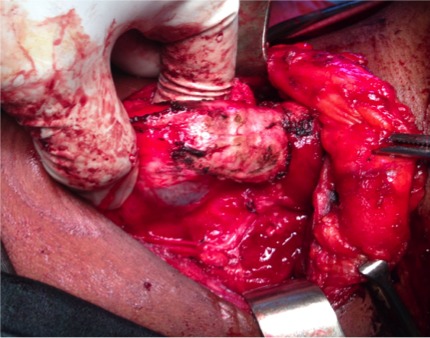
Ouverture trachéale incomplete

**Figure 3:**
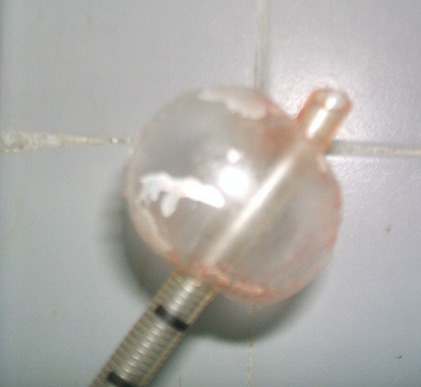
Hernie du ballonnet

Il s’agissait de trois patientes âgées de 27, 30 et 42 ans. Elles étaient admises au bloc opératoire pour une thyroïdectomie totale sous anesthésie générale indiquée devant un goitre multi nodulaire. La durée d’évolution du goitre variait entre 3 ans et 8 ans. Aucune des patientes ne présentait un antécédent pathologique particulier. Sur le plan anesthésique, elles étaient classées Cormack 2 pour l’une d’entre elles et Cormack 1 pour les deux autres. Nos 3 malades ont bénéficié d’une intubation orotrachéale avec une sonde N°7,5 munie d’un ballonnet. L’intubation a été facilitée par l’utilisation d’un guide rigide. Au cours de la chirurgie thyroïdienne, au moment de la section du ligament de Gruber, nous avons découvert une protrusion du ballonnet dans le champ opératoire (Figure 1 et Figure 2). Cette protrusion s’est faite à travers une effraction trachéale de siège postéro latérale dans les trois cas. A l’extubation, nous avons noté chez une des patientes, une hernie du ballonnet de la sonde d’intubation (Figure 3). Les découvertes per opératoires ainsi que notre attitude thérapeutique ont été résumées dans le Tableau 1. Il n’y avait pas de troubles ventilatoires ni de trachéomalacie. Les suites opératoires étaient simples.

**Tableau 1:** Découvertes et gestes

Patientes	Découvertes per opératoires	Attitude thérapeutique
CAS 1	Goitre nodulaire Effraction trachéale postéro latérale gauche (≈4cm)	Thyroïdectomie totale Suture de la brèche trachéale au fil résorbable Drainage par deux lames de Delbet
CAS 2	Goitre nodulaire Effraction trachéale postéro latérale droite (≈2,5cm) Hernie du ballonnet	Mêmes gestes
CAS 3	Goitre nodulaire plongeant avec déviation trachéale droite Plaie trachéale postéro latérale gauche incomplète (adventice intacte)	Thyroïdectomie totale Abstention chirurgicale sur la plaie trachéale Drainage aspiratif par un drain de Redon Jost

## Discussion

Les plaies trachéales post-intubations sont rares. Massimo et al rapportent 30 cas sur 12 ans [2]. Minambres E et al retrouvent une plaie trachéale pour 20000 intubations [3]. Le diagnostic peut être fait devant une détresse respiratoire, un emphysème sous cutané, une hémoptysie, une fuite persistante des drains, un pneumothorax chez un patient intubé ou dans les suites de l’extubation [1, 2]. La bronchoscopie au tube souple ou la tomodensitométrie visualisent la plaie et confirment le diagnostic.

Dans notre cas de chirurgie thyroïdienne, le diagnostic a été posé en per opératoire avec la découverte du ballonnet de la sonde d’intubation dans le champ opératoire. Il s’agit d’une situation spectaculaire. Plusieurs causes ont été décrites dans la littérature. Il s’agit des difficultés d’intubation, d’une déchirure de la membraneuse par le bec de la sonde d’intubation, par un guide rigide, un effort de toux avec ballonnet gonflé au réveil, un sur-gonflage du ballonnet [1, 2]. La pression du ballonnet n’a pas été mesurée dans notre étude faute de manomètre de pression. Cependant, la pression idéale du ballonnet est de 20 mmHg soit de 27 cm H2O. Au delà de cette pression, des complications trachéales à type de plaies et/ou de sténoses peuvent survenir. Un manomètre de pression doit ainsi faire partie intégrante du matériel d’intubation. La manipulation de la trachée au cours des chirurgies thyroïdiennes ainsi que la modification de l’anatomie et de la structure de la trachée dans les goitres anciens volumineux (déviation, trachéomalacie) sont aussi mis en cause. Nous avons noté un cas de déviation trachéale importante, dans notre étude, avec un goitre ancien, par contre aucun cas de trachéomalacie n’a été décelé. Nous avons aussi objectivé un cas de hernie du ballonnet qui est une déformation du ballonnet de la sonde d’intubation. Elle peut être due à un défaut de fabrication ou un recyclage abusif de la sonde d’intubation qui doit normalement être à usage unique. Elle pourrait être évitée en testant le ballonnet avant l’intubation. Il ressort de notre étude que les plaies trachéales prédominent chez les sujets de sexe féminin conformément à la littérature [1,2]. Ceci s’explique par une membrane postérieure plus fine chez la femme mais aussi par une surestimation du diamètre et de la taille de la trachée chez la femme [2].

Dans notre étude, les plaies trachéales siègent toutes au niveau de la jonction membrano-cartilagineuse. Rappelons que, la paroi antérieure de la trachée est cartilagineuse tandis que la paroi postérieure est membraneuse. Le raccordement entre les deux se fait au niveau postéro latéral par une bande musculaire lisse. Cette zone de jonction constitue une zone de moindre résistance où siège la majorité des ruptures trachéales. Il n’y a pas de consensus dans le traitement de ces plaies trachéales. A travers la littérature, le traitement varie d’une étude à l’autre. Prunet B et al optent pour un traitement chirurgical [4]. Selon Kaloud H et al, le traitement peut être médical ou chirurgical selon les caractères de la plaie trachéale [5]. Dans nos cas, nous préconisons avant la fermeture, une suture de la plaie trachéale au fil résorbable dans les cas d’ouverture complète de la paroi. Il faudra par la suite drainer par des lames de Delbet. Un drainage aspiratif risque de ne pas être efficace du fait des fuites d’air. Dans les cas d’ouverture incomplète de la paroi trachéale, un traitement médical anti-inflammatoire (corticothérapie en cure courte) en post opératoire sans geste chirurgical associé donne de bons résultats. Dans ces cas, le drainage peut être aspiratif. Dans tous les cas, une surveillance stricte du patient en post opératoire s’impose. La trachéotomie peut ainsi être évitée. Sa seule indication demeure une détresse respiratoire au réveil.

## Conclusion

Les plaies trachéales post-intubations sont rares et de découverte exceptionnelle en per opératoire lors de chirurgie cervicale. Eviter la manipulation intempestive de la trachée au cours des chirurgies cervicales permettrait de réduire l’incidence de ces plaies.

## Conflits d’intérêts

Les auteurs ne déclarent aucun conflit d'intérêts.
